# Traumatic cataract following high-intensity focused ultrasound eyelid procedure

**DOI:** 10.5935/0004-2749.2024-0360

**Published:** 2025-04-07

**Authors:** Carlos Augusto Moreira Neto, José Pereira do Rêgo Neto, Thiago Meister

**Affiliations:** 1 Hospital de Olhos do Paraná, Curitiba, PR. Brazil

High-intensity focused ultrasound (HIFU) is a noninvasive procedure commonly used for skin tightening. However, it has the potential to cause severe ocular complications^(1^,^2)^.

A 29-year-old female presented with complaints of “white spots” in her right eye’s visual field following an eyelid HIFU application. Slit-lamp examination revealed four small lens opacities located outside the visual axis. Anterior segment optical coherence tomography confirmed focal opacities in the lens (Figure 1).

**Figure:**
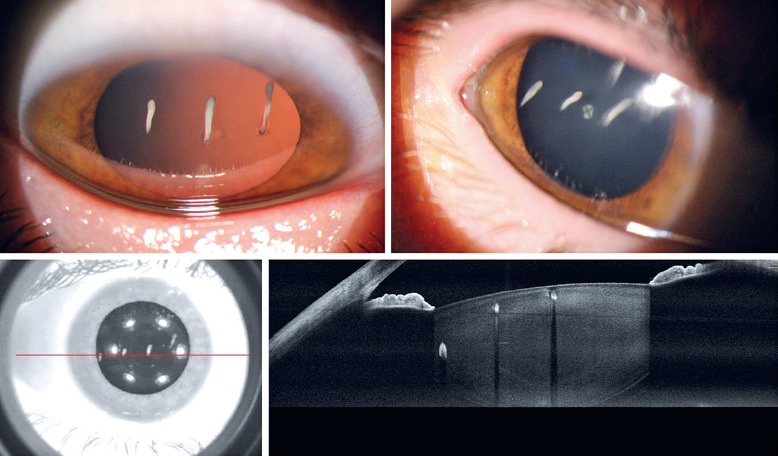

This case highlights the necessity for stringent ocular protection during HIFU procedures. Given the increasing popularity of HIFU for cosmetic applications, its risks may be underestimated^(3)^. Qualified practitioners must ensure patient safety by implementing appropriate protective measures.
